# Pollen Performance in *Clarkia* Taxa with Contrasting Mating Systems: Implications for Male Gametophytic Evolution in Selfers and Outcrossers

**DOI:** 10.3390/plants2020248

**Published:** 2013-04-24

**Authors:** Alisa A. Hove, Susan J. Mazer

**Affiliations:** 1 Biology Department, Warren Wilson College, P.O. Box 9000, Asheville, NC 28815, USA; 2 Department of Ecology, Evolution, and Marine Biology, University of California, Santa Barbara, CA 93106, USA; E-Mail: mazer@lifesci.ucsb.edu

**Keywords:** *Clarkia exilis*, *Clarkia unguiculata*, *Clarkia xantiana* ssp. *parviflora*, *Clarkia xantiana* ssp. *xantiana*, sexual selection, mating system evolution, pollen performance, pollen tube growth

## Abstract

We tested three predictions regarding the joint evolution of pollen performance and mating system. First, due to the potential for intense intrasexual competition in outcrossing populations, we predicted that outcrossers would produce faster-growing pollen than their selfing relatives. Second, if elevated competition promotes stronger selection on traits that improve pollen performance, then, among-plant variation in pollen performance would be lower in outcrossers than in selfers. Third, given successive generations of adaptation to the same maternal genotype in selfers, we predicted that, in selfing populations (but not in outcrossing ones), pollen would perform better following self- than cross-pollinations. We tested these predictions in field populations of two pairs of *Clarkia* (Onagraceae) sister taxa. Consistent with our predictions, one outcrosser (*C. unguiculata*) exhibited faster pollen germination and less variation in pollen tube growth rate (PTGR) among pollen donors than its selfing sister species, *C. exilis*. Contrary to our predictions, the selfing *C. xantiana* ssp. *parviflora* exhibited faster PTGR than the outcrossing ssp. *xantiana*, and these taxa showed similar levels of variation in this trait. Pollen performance following self- *vs.* cross-pollinations did not differ within either selfing or outcrossing taxa. While these findings suggest that mating system and pollen performance may jointly evolve in *Clarkia*, other factors clearly contribute to pollen performance in natural populations.

## 1. Introduction

The evolutionary and ecological factors that have promoted the diversity of plant sexual reproductive strategies have been a long-standing area of inquiry among evolutionary biologists. The evolutionary transition between outcrossing and selfing, in particular, has received considerable attention, in part because mating system shifts are widespread and have occurred independently within and across numerous taxa [1,2,3]. Interest in the evolutionary association between floral traits and mating system dates back to Darwin [4], who noted that self-fertilizing species often produce smaller and less conspicuous flowers than outcrossing congeners. Subsequent investigations have expanded upon Darwin’s work and found consistent differences between selfers and outcrossers in flower size [5,6,7,8], floral development rates [9,10], and pollen:ovule ratios [11,12,13,14]. The extent to which a species’ mating system influences its pollen performance, however, is not well understood (but see [15,16,17,18]). There are, however, several predictions concerning how plant mating systems should affect the strength, direction, and outcome of natural selection on pollen performance ([19] and described here). 

Pollen performance traits are often genetically based [20,21,22,23] and have been predicted to evolve in response to pollen competition if the pollen grains deposited onto stigmas are genetically variable with respect to pollen performance and their number routinely exceeds the number of ovules available for fertilization [24,25,26,27,28,29,30,31]. Because the haploid genotypes of pollen grains are expressed during the gametophyte stage, pollen competition may occur among different pollen donors as well as among the pollen grains of a single donor. Pollen competitive ability may be influenced by a variety of pollen characters, including pollen size and pollen grain volume [32]. Post-pollination processes, including the speed of pollen germination and pollen tube growth through the style, have also been shown to play a major role in determining the siring success of individual male gametophytes [33,34,35]. For example, when multiple pollen donors compete for access to ovules, rapid pollen tube growth through the style is positively correlated with siring success. Moreover, there is often strong overlap in gene expression between gametophytic and sporophytic life stages [36,37,38], suggesting that genes contributing to the success of high-quality gametophytes also enhance the fitness of the subsequent sporophyte generation. 

Evidence from studies conducted in natural populations suggests that pollen competition may regularly result in conditions favoring rapid pollen tube growth. First, although pollen limitation has been detected experimentally in many wild plant populations (reviewed by [39,40]), there are numerous plant species whose reproductive output is not limited by insufficient pollination (e.g., [41,42,43,44,45]). Even in populations in which mean seed production per fruit is pollen-limited, many individual flowers may receive more pollen grains than the number of ovules available for fertilization, and in these flowers selection favoring rapid pollen tube growth may be intense. Moreover, many wild plant species receive more pollen than they require to achieve full seed set [25,30,31,46], although intra-population and within-plant variation in pollen loads may reduce the consistency of pollen competition [47,48]. Second, multiple paternity, wherein the seeds produced by a single fruit are sired by multiple donors, has been reported in several outcrossing species [49,50,51,52,53,54,55], suggesting that selection among potential sires may occur when multiple pollen genotypes are deposited on stigmas if reproduction is not pollen-limited. 

Recently, we proposed three predictions regarding the evolution of pollen performance in predominantly self-fertilizing and outcrossing taxa [19]. Our predictions were based on the expectation that selfing and outcrossing taxa will differ in both the mean number of pollen genotypes deposited per stigma and the mean number of maternal plants to which individual sires are likely transfer their pollen. First, assuming that the stigmas of individual plants in highly outcrossing populations each receive on average a greater diversity of pollen genotypes than the stigmas of self-fertilizing individuals and that seed production per flower is not pollen-limited, there is a greater opportunity for selection among pollen genotypes in the styles of outcrossing taxa relative to closely related selfing taxa. Consequently, due to sustained selection in outcrossers over multiple generations for traits promoting the fertilization success of individual pollen grains, we predicted that pollen produced by outcrossing taxa should have evolved to germinate and/or to grow more rapidly through styles than the pollen of selfing sister taxa. Therefore, pollen from outcrossing taxa would exhibit faster germination and/or pollen tube growth than pollen from sister selfing taxa because of relatively relaxed selection on pollen performance in the latter since the time of evolutionary divergence. Moreover, if there are any physiological costs associated with rapid pollen tube growth, selfing taxa might even experience selection favoring slower growth. This prediction should also be considered in light of differences between outcrossers and selfers in floral size. Because outcrossers often produce larger flowers than their selfing relatives, their styles are also likely to be longer (e.g., [9,56]). In order to achieve fertilization success, the pollen tubes of outcrossers may travel a greater distance than their selfing counterparts, meaning that differences between selfers and outcrossers in pollen tube growth rates might not correspond to differences in how quickly pollen tubes reach the style base, particularly in cases where outcrossers have much longer styles than their selfing sister taxa. If pollen performance has evolved to be sufficiently accelerated in outcrossers and selection on pollen performance traits has relaxed in selfers, however, then we would also predict that the pollen tubes of outcrossers to travel a greater proportion of the style length per unit time than the pollen tubes of their selfing counterparts. 

Second, because selfing reduces the number of distinct pollen genotypes deposited on individual stigmas, the comparatively stronger opportunity for selection on pollen performance traits in outcrossing taxa should have purged variation in these traits more effectively in outcrossers relative to selfers. Accordingly, we predicted that outcrossing taxa would harbor less genetically based variation among individual pollen donors in pollen performance traits than closely related selfing taxa. This prediction is based on the expectation that intense pollen competition among pollen genotypes will result in selection favoring rapid pollen germination and/or growth in outcrossing taxa. It should also be noted that maternal plant identity and environmental factors such as temperature and resource availability may also contribute to among-donor variation in pollen tube growth and germination in outcrossing taxa [57,58,59,60,61,62,63,64,65] and that the intensity of pollen competition may vary within and among flowering seasons [40]. Consequently, evidence of past purging of genetic variation may be difficult to detect. See [19] for a detailed discussion of mechanisms that may contribute to the maintenance, or to the purging, of genetic variation in pollen performance.

Third, we predicted that in selfing taxa, potentially beneficial epistatic interactions (*i.e*., combinations of alleles at different loci that result in particularly high performance) that influence pollen performance are likely to be reliably and consistently inherited over multiple generations and thus accumulate in populations. In other words, because the pollen of selfers experiences successive generations of pollen germination and pollen tube growth in genetically consistent maternal plant tissue, their pollen should evolve to be particularly well adapted to grow within its parental genotype. By contrast, because outcrossers typically mate with multiple, genetically distinct partners over their lifetimes, such beneficial epistatic interactions are more likely to break down due to recombination, and are therefore less likely to remain stable in outcrossing taxa than selfing taxa. Moreover, outcrossers should experience strong selection favoring the ability of pollen to perform well in a wide variety of stigmatic and stylar genotypes, while selection favoring such tolerance may be comparatively weak in autogamous selfers, whose pollen grains rarely germinate on foreign maternal genotypes. Consequently, we hypothesized that in regularly selfing taxa, pollen should have evolved to perform better following self-pollinations than following cross-pollinations. By contrast, in habitually outcrossing taxa, we predicted that pollen would either perform better following cross-pollinations than self-pollinations (e.g., [66]) or that the pollen source (cross or self) would not significantly influence pollen performance (e.g., [17,67,68]).

Previous research conducted on genotypes sampled from selfing and outcrossing *Clarkia tembloriensis* (Onagraceae) populations and cultivated in growth chambers provides preliminary support for our predictions. Smith-Huerta [17] and Kerwin and Smith-Huerta [15] compared *in vivo* pollen tube growth rates (PTGR) between two *C. tembloriensis* populations (one highly selfing and one highly outcrossing). Both investigations found that pollen from the outcrossing population tended to grow more quickly than pollen from the selfing population. This difference between selfing and outcrossing populations was observed following both cross-pollinations and self-pollinations of individual pollen donors. To date, however, very few published studies have compared pollen performance between selfers and outcrossers in multiple populations under natural conditions where ecological factors such as pollinator service and climate may also influence the evolution of pollen performance traits (but see [18]). 

In recent decades, the annual wildflower genus *Clarkia* has been the subject of extensive investigation of the evolution and ecology of mating system transitions (e.g., [9,10,69,70,71,72,73,74]). Within the genus, selfing has evolved independently from outcrossing numerous times [56,75,76]. Here we investigate the correlated evolution of pollen performance traits and mating system in two pairs of predominantly outcrossing and selfing sister taxa that occur in the southern Sierra Nevada, California: the pollinator-dependent outcrosser *Clarkia xantiana* ssp. *xantiana* Gray (hereafter “*xantiana*”) and its selfing sister subspecies, *C. xantiana* ssp. *parviflora* (Eastw.) H. Lewis and Raven (hereafter “*parviflora*”), as well as the outcrosser *C. unguiculata* Lindley (hereafter “*unguiculata*”) and its selfing sister species *C. exilis* H. Lewis and Vasek (hereafter “*exilis*”). We address the following questions: (1) Does pollen from outcrossing taxa exhibit faster pollen germination and faster pollen tube growth rates (PTGR) than pollen from closely related selfing taxa in field populations? An observation of slower pollen germination or pollen tube growth in selfers than outcrossers would provide evidence in support of the hypothesis that, since the divergence between sister taxa, selection favoring rapid pollen performance traits in selfers has weakened relative to its strength in their outcrossing counterparts. Alternatively, in the absence of strong gametophytic selection (*i.e.*, in the selfers), if there is a fitness cost associated with rapid pollen germination or pollen tube growth, then selection may have favored lower rates of these traits in selfers relative to their outcrossing progenitors; (2) Do outcrossing taxa exhibit less phenotypic variation among individuals in pollen performance traits than their selfing sister taxa in field populations, consistent with the prediction that selection has more effectively purged variation in pollen performance in outcrossers due to more intense gametophytic competition among genotypes? and (3) Does pollen from selfing taxa (*exilis* and *parviflora*) exhibit faster growth following self-pollination than following cross-pollination? To address these questions, we evaluated three pollen performance traits in each taxon: pollen germination (a categorical variable indicating whether pollen tubes had penetrated the style), PTGR (the mean speed by which pollen tubes travel through the style), and the proportion of the entire style length traveled per hour (Table 1). Prior to comparing sister taxa with respect to the latter two traits, we controlled statistically for the effect of temperature on pollen tube growth. See the *Experimental Section* for a detailed description of how traits were measured and compared between sister taxa. 

## 2. Results and Discussion

### 2.1. Pollen Germination in Taxa with Contrasting Mating Systems

The relative frequencies of stigmas with germinated and non-germinated pollen grains differed significantly between the sister species *unguiculata* and *exilis* following cross-pollinations (Table 2). Germination failure was more common in the selfing species, *exilis*, than in its outcrossing counterpart *unguiculata* (Fisher’s Exact Test, *p* < 0.0001, n = 439). At least one pollen tube was observed in the style following 92.2% of *unguiculata* cross-pollinations. By contrast, only 34.8% of *exilis* cross-pollinations resulted in the presence of at least one pollen tube in the style 2.5 hours post-pollination. We also observed this difference following self-pollinations; pollen germination was observed following 83.9% of *unguiculata* self-pollinations, but only 24.1% of *exilis* self-pollinations. Moreover, *exilis* pollen tubes were significantly less likely to be observed at the stigma-style junction than *unguiculata* pollen tubes (Fisher’s Exact Test, *p* < 0.0001, n = 85).

The sister subspecies *xantiana* and *parviflora* showed a different pattern (Table 2). The likelihood of observing a pollen tube at the stigma-style junction did not differ between taxa following cross-pollinations (Fisher’s Exact Test, *p* = 0.07, n = 513) or self-pollinations (Fisher’s Exact Test, *p* = 0.53, n = 148). 

**Table 1:** Pollen performance traits measured for this study and methods used to determine trait values. *L_s_ =* style length (mm); *C_x,y_* = number of callose plugs present in the interval between *x* and *y*, where *x* = distance from the stigma-style junction (mm); *y = x + 1 mm*; *C_0,1_* = number of callose plugs present 0–1 mm from the stigma-style junction; *t* = time between pollination and style harvest (hours). See the *Experimental Section* for additional details.

Trait	Definition	Determination of trait value	Styles used to determine trait value
**Pollen germination**	A binary variable signifying pollen grain germination and initial pollen tube entry into the style.	We concluded that pollen germination had occurred if we observed at least one pollen tube in the stigma-style junction 2.5 hours following pollination.	All styles sampled
**Pollen tube growth rate, PTGR** **(mm/hour)**	Mean speed by which pollen tubes travel through the style	1. Estimate mean pollen tube length (mm), *L_p_* 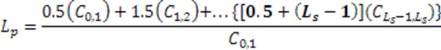	Styles in which at least one pollen tube was observed at the stigma-style junction 2.5 hours post-pollination
		2. Divide by *t* to estimate pollen tube growth rate (PTGR) 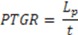	
**Proportion of the entire style length traveled per hour**	Mean proportion of the style length traversed per hour	1. Divide PTGR by the length of the style in which it was estimated. 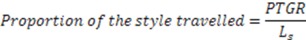	Styles in which at least one pollen tube was observed at the stigma-style junction 2.5 hours post-pollination

**Table 2:** Pollen germination observed in each study population following cross-pollinations and self-pollinations. The column “>0 tubes observed” shows the number of styles in which at least one pollen tube was observed within 1mm of the stigma-style junction. The column “0 tubes observed” shows the number of styles in which no pollen tubes were observed. % Germination success refers to the percentage of styles in which at least one pollen tube was observed 2.5 hours post-pollination.

Taxon	Population	Type of pollination	>0 tubes observed	0 tubes observed	% Germination success
***exilis***	Stark Creek	Cross	27	77	26.0%
		Self	7	22	24.1%
	Willow Spring	Cross	28	26	51.9%
		Self	*n/a*	*n/a*	
	Populations pooled	Cross	55	103	34.8%
***unguiculata***	Jack and Stage	Cross	113	12	90.4%
		Self	16	5	76.2%
	Live Oak	Cross	146	10	93.6%
		Self	31	4	88.6%
	Populations pooled	Cross	259	22	92.2%
		Self	47	9	83.9%
***parviflora***	Long Valley	Cross	130	1	99.2%
		Self	37	2	94.9%
	Wofford Heights	Cross	134	8	94.4%
		Self	12	3	80.0%
	Populations pooled	Cross	264	9	96.7%
		Self	49	5	90.7%
***xantiana***	Borel Road	Cross	116	3	97.5%
		Self	31	4	88.6%
	Camp 3	Cross	107	14	88.4%
		Self	57	2	96.6%
	Populations pooled	Cross	223	17	92.9%
		Self	88	6	93.6%

### 2.2. Pollen Tube Growth and Phenotypic Variation in Taxa with Contrasting Mating Systems

#### 2.2.1. Cross-Pollinations

**Figure 1:**
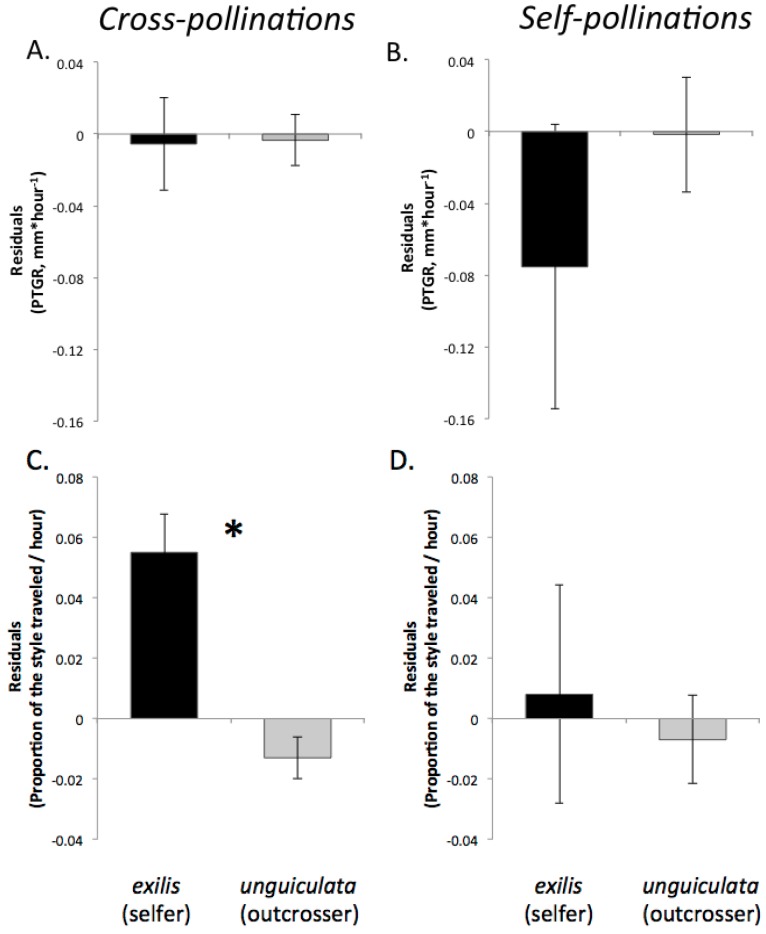
Temperature-adjusted species means (±1SE) for *exilis* (a selfer, black bars) and *unguiculata* (an outcrosser, grey bars) for pollen performance traits following cross-pollinations and self-pollinations conducted in field populations during spring 2008. (**A**) and (**B**): pollen tube growth rate (PTGR). (**C**) and (**D**): proportion of the entire style length traversed by pollen tubes that entered the style within 2.5 hours of pollination. An asterisk (*) indicates cases where a given pollen performance trait differs between sister species (*p* < 0.05).

Temperature-adjusted PTGR following cross-pollinations did not differ between *exilis* and *unguiculata* (Table 3, Figure 1A). Sister species differed, however, with respect to style length; on average *exilis* styles were significantly shorter than *unguiculata* styles (one-way ANOVA, F_1,522_ = 2156.3, *p* < 0.0001). Mean style length was 10.87 mm (SE = 0.09) in *exilis* populations and 18.9 mm (SE = 0.13) in *unguiculata* populations. Our comparison of the proportion of the style length traveled revealed that, even though *exilis* pollen germinated less frequently than *unguiculata* pollen within 2.5 hours of pollination, due to *exilis*’ shorter styles, pollen tubes traversed a greater proportion of the style length in *exilis* than in *unguiculata* (Table 3, Figure 1C).

**Table 3:** Mixed model nested ANOVAs comparing mean temperature-adjusted pollen performance traits between the sister species *exilis* and *unguiculata* following cross- and self-pollinations. In these analyses, *p-*values < 0.05 are associated with statistically significant fixed effects. We used the restricted maximum likelihood (REML) method to estimate variance components for the random effects of Pollen Recipient and Pollen Donor. Random effects associated with variance components whose 95% confidence intervals (CI) do not include zero are statistically significant and indicated in bold italics.

Type of pollination	Trait	Source	df_N,D_	*F*-ratio or Variance Component	*p*-value or 95% CI
**Cross**	PTGR	Taxon	1, 115	0.005	0.944
		Recipient[Taxon]		0.007	***0.002–0.011***
		Donor[Taxon, Recipient]		0.003	−0.002-0.008
	Proportion of the entire style length traveled	Taxon	1, 100.7	**22.23**	***<0.0001***
		Recipient[Taxon]		0.001	***0.000–0.003***
		Donor[Taxon, Recipient]		0.001	***0.000–0.003***
**Self**	PTGR	Taxon	1, 40.8	0.7448	0.3932
		Recipient[Taxon]		0.013	−0.008–0.034
		Donor[Taxon, Recipient]		0.000	0.000–0.000
	Proportion of the entire style length traveled	Taxon	1, 40.5	0.1492	0.7013
		Recipient[Taxon]		0.002	***0.000–3.460***
		Donor[Taxon, Recipient]		0.001	***0.001–0.003***

**Figure 2:**
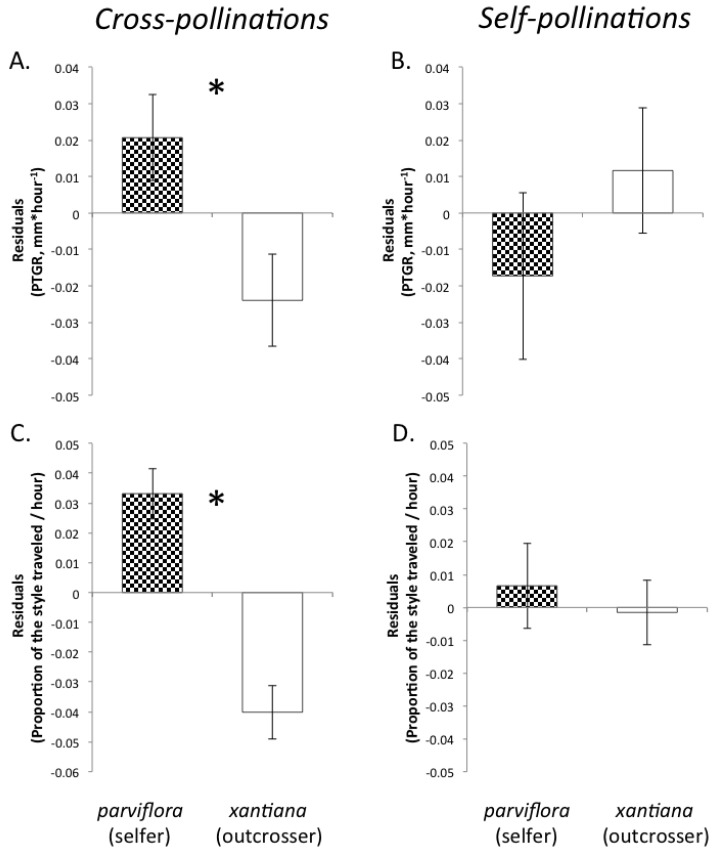
Temperature-adjusted subspecies means (±1SE) for *parviflora* (a selfer, checkered bars) and *xantiana* (an outcrosser, white bars) for pollen performance traits following cross-pollinations and self-pollinations conducted in field populations during spring 2008. (**A**) and (**B**) pollen tube growth rate (PTGR). (**C**) and (**D**) proportion of the entire style length traversed by pollen tubes that entered the style within 2.5 hours of pollination. An asterisk (*) indicates cases where a given pollen performance trait differs between sister sub-species (*p* < 0.05).

Contrary to our initial expectation, we found that pollen derived from the selfer *parviflora* grew more rapidly than pollen from *xantiana*. Accounting for the effect of temperature on pollen tube growth, *parviflora* exhibited faster PTGR (Table 4, Figure 2A) and traversed a greater proportion of style length than *xantiana* (Table 4, Figure 2C). Similar to the *exilis*/*unguiculata* comparison, *parviflora* styles (mean ± SE = 11.76 ± 0.17) were shorter than *xantiana* styles (mean ± SE = 15.29 ± 0.17) (F_1,659_ = 212.52, *p* < 0.0001), contributing to higher proportional distance traveled by *parviflora* pollen tubes.

**Table 4:** Mixed model nested ANOVAs comparing mean temperature-adjusted pollen performance traits between the sister subspecies *parviflora* and *xantiana* following cross- and self-pollinations. In these analyses, *p-*values < 0.05 are associated with statistically significant fixed effects. We used the restricted maximum likelihood (REML) method to estimate variance components for the random effects of Pollen Recipient and Pollen Donor. Random effects associated with variance components whose 95% confidence intervals (CI) do not include zero are statistically significant and indicated in bold italics.

Type of pollination	Trait	Source	df_N,D_	*F*-ratio or Variance Component	*p*-value or 95% CI
**Cross**	PTGR	Taxon	1, 119.5	6.620	***0.0113***
		Recipient[Taxon]		0.005	***0.003–0.008***
		Donor[Taxon, Recipient]		0.000	−0.002–0.002
	Proportion of the entire style length traveled	Taxon	1, 121.5	35.69	***<0.0001***
		Recipient[Taxon]		0.003	***0.002–0.005***
		Donor[Taxon, Recipient]		0.000	***0.000–0.001***
**Self**	PTGR	Taxon	1, 89.3	1.019	0.3155
		Recipient[Taxon]		0.007	***0.001–0.013***
		Donor[Taxon, Recipient]		0.000	***0.000–0.000***
	Proportion of the entire style length traveled	Taxon	1, 95.9	0.247	0.6206
		Recipient[Taxon]		0.001	***0.000–1.263***
		Donor[Taxon, Recipient]		0.003	***0.002–0.004***

**Figure 3:**
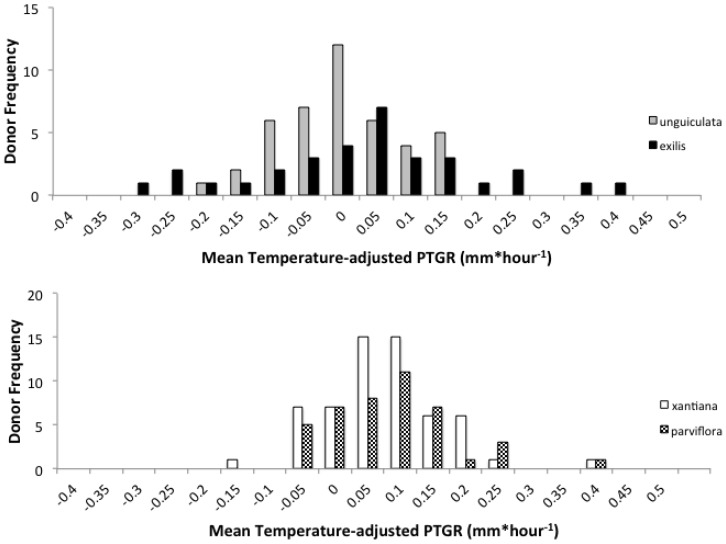
Frequency distribution showing mean temperature-adjusted PTGR for *unguiculata* (grey) and *exilis* (black) pollen donors (upper panel) and *xantiana* (white) and *parviflora* (checkered) pollen donors (lower panel). Each bar corresponds to the number of pollen donors whose mean PTGR fell within the range indicated on the X-axis.

Differences between sister taxa in the magnitude of phenotypic variation in pollen performance traits depended on the sister taxon pair. *Exilis* and *unguiculata* populations differed significantly in levels of phenotypic variation in PTGR and in the proportion of the style length traveled in a manner that was consistent with our initial prediction (Figure 3, Table 5). Both populations of the outcrosser, *unguiculata*, exhibited significantly *lower variance* in PTGR than *exilis* populations. Moreover, the Stark Creek *exilis* population exhibited significantly *higher variance* in PTGR and in the proportion of the style length traveled than any of the other *unguiculata* or *exilis* populations surveyed. By contrast, populations of *parviflora* and *xantiana* exhibited similar variances in PTGR and the proportion of the style length traveled (Figure 3, Table 5).

#### 2.2.2. Self-Pollinations

We did not detect any statistically significant differences in pollen performance between either pair of sister taxa following self-pollinations. Mean pollen tube growth rates were not statistically distinguishable between *unguiculata* and *exilis* (Table 3, Figure 1B) due to the particularly high variation in PTGR exhibited by both taxa. Similarly, the pollen tubes of the sister species traversed a similar proportion of the style length, even though the styles of *exilis* are shorter than those of *unguiculata* (Table 3, Figure 1D). Likewise, these traits did not differ between *xantiana* and *parviflora* following self-pollinations (Table 4, Figure 2B,D).

### 2.3. Pollen Performance Following Self- *vs.* Cross-Pollinations within Taxa

We did not observe a significant association between pollination type and the frequency of germination success (determined by the presence of at least one pollen tube at the stigma-style junction 2.5 hours post-pollination) in any of our focal taxa. Germination success was as likely to occur following cross- and self-pollination in *exilis* (Fisher’s Exact Test, *p* = 1.000, n = 133) and its sister species *unguiculata* (Fisher’s Exact Test, *p* = 0.07, n = 337). This was also the case in *parviflora* (Fisher’s Exact Test, *p* = 0.063, n = 327) and *xantiana* (Fisher’s Exact Test, *p* = 1.000, n = 334). We observed a similar pattern when we compared the speed of pollen tube growth through the style following self- and cross-pollinations within each of the four focal taxa. The average performance of individual pollen donors, measured as PTGR and the proportion of the style traveled per hour, was similar following cross-pollinations and self-pollinations (Table 6).

**Table 5:** Mean temperature-adjusted PTGR and the proportion of the style length traveled per hour, and associated standard deviation (SD) and standard error values (SE) for populations of the *Clarkia* sister taxa *exilis* and *unguiculata*, and ssp. *parviflora* and *xantiana.* Within each population, individual donor means were calculated across all recipient flowers following cross-pollinations. Population means were then calculated as the mean of the donors’ means for each pollen performance trait. Population variances were then compared among populations of sister taxa using the default tests in JMP 9.0 (O’Brien, Brown-Forsythe, Levene, and Bartlett tests). If differences were detected by these tests, the analysis of means for variance test [Levene’s Analysis of Means for Variances (ANOM) for variances] was used to identify populations whose absolute deviations from the median differed from the average cross-group absolute deviation from the median. Within each pair of sister taxa, population standard deviations whose corresponding variances are significantly higher (H) or lower (L) than the cross-population median of the variances (Levene’s ANOM, α = 0.05) are indicated in bold.

Taxon	Population	n	PTGR				Proportion of the style length traveled		
			Mean	SD	SE		Mean	SD	SE
***exilis***	Stark Creek	16	−0.0260	**0.1714 ^H^**	0.0429		0.0456	**0.1107 ^H^**	0.0277
	Willow Spring	13	0.0268	0.1671	0.0463		0.0701	0.1004	0.0279
***unguiculata***	Jack and Stage	20	−0.0732	**0.0680 ^L^**	0.0152		−0.0408	0.0274	0.0061
	Live Oak	22	0.0384	**0.0682 ^L^**	0.0146		0.0015	0.0301	0.0064
***parviflora***	Long Valley	20	−0.0015	0.0757	0.0169		−0.0293	0.0327	0.0073
	Wofford Heights	18	0.0530	0.0453	0.0107		0.0978	0.0319	0.0075
***xantiana***	Borel Rd	20	−0.0230	0.0607	0.0136		−0.0215	0.0302	0.0068
	Camp 3	21	−0.0229	0.0583	0.0127		−0.0524	0.0282	0.0062

**Table 6:** Nested ANOVAs evaluating the random effects of Pollen Donor and Pollination Type (cross or self) nested within Pollen Donor on temperature-adjusted pollen performance traits in *exilis* (Stark Creek population), as well as in *unguiculata*, *parviflora*, and *xantiana* populations. We used the restricted maximum likelihood (REML) method to estimate variance components for each taxon. Effects associated with variance components whose 95% confidence intervals (CI) do not include zero are statistically significant and are indicated in bold italics.

Taxon	Effect	Pollen tube growth rate (mm/hour)				Proportion of the style length traveled		
		Variance Component	95% CI	% of Total		Variance Component	95% CI	% of Total
***exilis***	Pollen Donor	0.0134	−0.0166–0.0434	38.66		0.0078	−0.0017–0.0173	58.83
	Pollination Type[Pollen Donor]	0.0022	−0.0277–0.0322	6.48		−0.0020	−0.0106–0.0067	−14.83
	Error	0.0190	0.0100–0.0499	54.86		0.0074	0.0038–0.0198	55.99
	Total	0.0347		100		0.0132		100
***unguiculata***	Pollen Donor	***0.0049***	***0.0013–0.0085***	18.52		***0.0009***	***0.0002–0.0016***	16.86
	Pollination Type[Pollen Donor]	−0.0010	−0.0044–0.0024	−3.71		−0.0002	−0.0009–0.0004	−4.48
	Error	0.0225	0.0189–0.0272	85.19		0.0046	0.0038–0.0055	87.62
	Total	0.0264		100		0.0052		100
***parviflora***	Pollen Donor	***0.0035***	***0.0005–0.0064***	16.74		***0.0042***	***0.0015–0.0069***	39.24
	Pollination Type[Pollen Donor]	−0.0007	−0.0033–0.0018	−3.52		0.0008	−0.0008–0.0023	7.16
	Error	0.0179	0.0152–0.0215	86.78		0.0057	0.0048–0.0069	53.61
	Total	0.0207		100		0.0107		100
***xantiana***	Pollen Donor	0.0015	−0.0007–0.0036	8.22		0.0004	−0.0001–0.0008	9.42
	Pollination Type[Pollen Donor]	−0.0007	−0.0029–0.0014	−4.16		−-0.0002	−0.0006–0.0003	−4.45
	Error	0.0173	0.0145–0.0209	95.94		0.0038	0.0032–0.0045	95.03
	Total	0.0180		100		0.0040		100

### 2.4. Gametophytic Divergence in Taxa with Contrasting Mating Systems

The results reported here provide inconsistent support for the hypothesis that, similar to other primary and secondary sexual traits that have been previously investigated, pollen performance traits diverge in a predictable way concurrently with or following evolutionary transitions in mating system. The extent to which pollen performance traits evolve in conjunction with mating system has not been well-studied [19]. Our observations of pollen germination rates among flowers of the sister species *unguiculata* and *exilis* are consistent with those reported by previous studies of pollen performance in populations or taxa with contrasting mating systems (e.g., [15,17]). Recently, Taylor and Williams [18] also detected a difference in PTGR between selfing and outcrossing taxa in field populations; they found that the dioecious, obligate outcrosser, *Trithuria austinensis* had faster *in vivo* PTGR than its hermaphroditic, highly selfing relative, *T. submerse*.

As we initially predicted, pollen performance differed between the sister species *unguiculata* and *exilis.* Although the sister taxa exhibited similar pollen performance within the style, sister taxa differed dramatically in the relative frequencies of stigmas on which pollen had *vs*. had not germinated. *Clarkia exilis* pollen tubes were observed much less frequently in the upper style 2.5 hours after pollination than *unguiculata* pollen tubes. Low levels of pollen germination in *exilis* may have been observed because stigmas were not receptive at the time of pollination. This possibility, however, seems unlikely because in all four taxa we took care to pollinate stigmas only when they were fully unfurled and covered with papillate hairs. Previous pollinations of unfurled, papillate stigmas in all four focal taxa in pollinator-free greenhouses have resulted in nearly 100% fruit set. Moreover, if our visual assessments of stigma receptivity were erroneous, we would have expected to have observed low pollen germination in all four focal taxa; *exilis* was the only taxon in which we observed no pollen tubes at the stigma-style junction 2.5 hours post-pollination in the majority of our styles (65.1% of *exilis* styles with zero pollen tubes observed, Table 2). An alternative possibility is that because pollen from selfing taxa does not require pollinator transport to receptive stigmas, individual pollen grains of selfers may be developmentally less mature than the pollen of outcrossers at the time of apparent stigma receptivity (when the pollinations were conducted). If so, these immature pollen grains may simply require more time to complete their maturation and to germinate than does the pollen from outcrossers. If this were the case, however, we would expect to have observed slower germination in both *exilis* and *parviflora* relative to their outcrossing counterparts, and *parviflora* did not exhibit this delay.

We observed significant differences between *exilis* and *unguiculata* with respect to pollen germination. We scored pollen germination success as a binary variable, which provides a rough estimate of the amount of pollen germinated in both selfers and outcrossers because pollinations resulting in at least one pollen tube entering the style were grouped with pollinations resulting in dozens of pollen tubes entering the style. We therefore recommend that future studies of these sister species (and other pairs of sister taxa) evaluate germination success use a more precise measure of pollen germination, such as the proportion of pollen grains that germinate post-pollination. 

Our findings with respect to *xantiana* and *parviflora*, however, contrast with the observations reported in other comparative studies of pollen performance in outcrossing and selfing populations or taxa (e.g., [15,17]) and suggest that other ecological factors may often overwhelm the selective pressures exerted by mating system on the evolution of pollen performance traits, either by imposing direct environmental effects on pollen performance or by exerting other selective pressures. In a companion study conducted over three flowering seasons, we found that insufficient pollination frequently limited seed production at the individual flower level in *xantiana* populations (Hove, Mazer, and Ivey, in preparation). Previous work has also detected pollen limitation in *xantiana* populations occurring in the Lake Isabella region [77,78]. By contrast, over a three-year period, we detected no evidence of pollen limitation in *unguiculata* populations. This suggests that the intensity of pollen competition may differ consistently between *xantiana* and *unguiculata*. If pollen limitation occurs frequently in *xantiana* populations, then selection among potential pollen donors or pollen genotypes favoring rapid pollen germination and/or pollen tube growth would be relatively weak in this taxon. It should be noted, however, that pollen competition may occur even in pollen-limited populations. For example, even if reproduction at the whole plant level is pollen limited, many individual flowers may still receive pollen loads sufficiently large to induce pollen competition for access to their ovules. Further studies of variation in pollen deposition within and among plants (c.f. [30,31]) are needed to in order to determine whether the magnitude of pollen competition differs between *xantiana* and *unguiculata* populations.

Although intense competition among diverse pollen genotypes is predicted in outcrossing taxa, it is also reasonable to speculate that the strength of selection on pollen performance traits will be influenced by the timing of and the quantity of pollen deposited on the stigma. In a series of studies conducted in Mediterranean field populations of multiple species in the mint family (Lamiaceae), Herrera [30,31] found extensive within- and among-plant variation in both pollen loads and the number of pollen tubes within the flower styles. On average, pollen tube numbers varied more within plants than it did among plants at the same site, although the number of pollen tubes exceeded the number of ovules in most flowers studied. One interpretation of Herrera’s observations is that unpredictable variation in competitive environments encountered following pollen deposition may dramatically reduce the opportunity for selection among pollen donors when averaged over all available stigmas and styles. 

It is also possible that differences in divergence time between the two pairs of sister taxa underlie the contrasting patterns that we observed. While estimates of the time of divergence are lacking for *exilis* and *unguiculata*, the fact that the sister species do not produce viable interspecific hybrids [56] may indicate a relatively ancient divergence. By contrast, a recent study of the divergence between the subspecies *parviflora* and *xantiana* concluded that they have diverged relatively recently (roughly 65,000 years ago) and that they can form viable hybrids following artificial crosses (see [79,80] for evidence that sister subspecies are cross compatible). On the other hand, the differences observed in the PTGR of conspecific populations of *C. tembloriensis* that differed in mating system [17] suggest that pollen performance traits can evolve rapidly.

### 2.5. Phenotypic Variation in Pollen Performance Traits

Our comparison of variances between *unguiculata* and *exilis* provides support for the prediction presented by Mazer *et al*. [19] that outcrossing and selfing taxa should harbor different levels of genetic variation in pollen performance traits. This prediction was based on the expectation that the intensity of competition among distinct pollen genotypes for access to a limited number of ovules should routinely differ between selfers and outcrossers. Consistent with the prediction that pollen competition is more likely to reduce genetically based variation in pollen performance traits in outcrossing taxa, *unguiculata* populations exhibited *less* phenotypic variation in PTGR among pollen donors than *exilis* populations. 

By contrast, the similarity in phenotypic variances in pollen tube growth rates among populations of *xantiana* and *parviflora*, in conjunction with the observation of pollen limitation in *xantiana* populations [77,78], is consistent with the interpretation that pollen competition in *xantiana* populations may not be strong or consistent enough to promote sustained directional selection on pollen performance traits, and thereby to purge variation in such traits. Alternatively, the time of divergence between these two subspecies may be so recent that insufficient time has passed to generate a phenotypic divergence between them with respect to pollen performance. Environmental variation within and among populations may also have made it difficult to detect differences in phenotypic variance between *xantiana* and *parviflora* field populations. The speed by which pollen tubes germinate and travel through styles can be influenced by several environmental factors, including temperature, soil pH, herbivory, and nutrient availability [22,57,59,62]. For example, Smith-Huerta *et al.* [81]. Compared PTGR of individual *unguiculata* pollen donors on maternal plants watered with a nutrient solution *versus* maternal plants watered with distilled water. They found that pollen donors generally grew more rapidly through styles of plants watered with distilled water, and suggested that low nutrient status may hinder maternal plant’s ability to discriminate among potential sires. 

We attempted to control for environmental effects on pollen tube growth by comparing temperature-adjusted residuals between taxa. Using this approach we were able to detect differences in phenotypic variation among pollen donors between one pair of sister taxa (*exilis* and *unguiculata*). Nevertheless, given the potential influence of environmental conditions experienced by paternal and maternal sporophytes in field populations on the phenotypic expression of pollen grains, it is important to clarify that the genetic basis of the traits studied here (or their variances) is unknown. Future investigations conducted in a common environment and designed specifically to estimate additive genetic variation in PTGR are necessary to determine whether sister taxa harbor different levels of genetic variation in pollen performance.

### 2.6. Pollen Performance Following Cross- *vs.* Self-Pollinations within Selfing Taxa

We initially predicted that, due to repeated generations of pollen germination and pollen tube growth in a consistent stylar environment, the pollen produced by selfing taxa, such as *exilis* and *parviflora*, would be particularly well-adapted to its own parental genotype. We specifically predicted that within selfing populations, pollen donors would exhibit greater germination success and faster PTGR following self-pollinations relative to cross-pollinations. This prediction, however, was not supported by the results of this study. Pollen donors from both selfing taxa had similar levels of germination success, similar PTGR, and similar proportional growth through the style following self- and cross-pollinations. The pollen of these selfing taxa appears to be unaffected by exposure to novel stigmatic genotypes. 

### 2.7. Differences in Pollen Performance between Xantiana and Parviflora: Other Ecological Considerations

The *Clarkia* sister taxa studied here differ phenotypically in numerous life-history, floral, and physiological traits that may be influence plant fitness. The indirect influence of such traits on pollen performance (in combination with pollen limitation in *xantiana* populations) could contribute to the faster PTGR within the styles of *parviflora* pollen compared to *xantiana* pollen. The selfer, *parviflora*, occupies hot, arid habitats and generally flowers and produces seeds earlier and more rapidly than *xantiana*. Runions and Geber [9] found that *parviflora* consistently exhibits faster floral development rates than *xantiana*. More recently, Mazer *et al*. [70] reported that *parviflora* exhibits faster rates of photosynthesis and transpiration prior to the onset of flowering and during flowering than *xantiana*. Prior to flowering *parviflora* also has lower instantaneous water use efficiency than *xantiana*. 

If pollen performance traits in *parviflora* are genetically correlated with physiological or floral developmental traits under natural selection, then rapid pollen tube growth may evolve as part of a general life history strategy involving fast, efficient reproduction. Physiological condition has been widely predicted to affect the evolution of sexually selected traits in animals [82]. The extent to which the expression of sexually selected traits in plants is associated with the speed by which individuals must complete their life cycle or by individual physiological condition, however, remains to be seen.

## 3. Experimental Section

### 3.1.Study Taxa

The two pairs of *Clarkia* sister taxa evaluated here (*unguiculata vs*. *exilis*, and *xantiana* *vs*. *parviflora*) have been the focus of extensive research on the evolutionary ecology of mating system transitions. Previous research has demonstrated that these sister taxa have diverged with respect to flower size (including style length) [56,80], pollen:ovule ratios [12,69], flowering phenology [10,71,83], floral development rates [9,10], and gas exchange physiology [70,84]. On average, the selfers, *exilis* and *parviflora*, produce smaller flowers; shorter styles; have lower pollen:ovule ratios, lower pollen production, and higher ovule production; flower earlier; and develop more rapidly than their outcrossing relatives. The pollen grains produced by selfing and outcrossing sister taxa are of similar size and visually indistinguishable from one another (A. Hove, personal observation). 

The mating system divergence between sister taxa is facilitated by differences in spatial separation (herkogamy) and temporal separation (dichogamy) of anthers and receptive stigmas. The flowers of *unguiculata* and *xantiana* generally exhibit strong herkogamy and protandry (*i.e.*, anthers begin shedding pollen several days before stigmas mature). As the globose stigmas become receptive, they unfold to expose a flattened, recurved four-lobed surface, which is covered with papillae to which pollen grains readily adhere. By contrast, in the flowers of autogamous taxa, *exilis* and *parviflora*, stigmas generally mature and become receptive in close physical proximity to dehiscent anthers [10,80,85,86]. 

All four focal taxa occur in the southern Sierra Nevada, California, USA [87]. In this region, *xantiana*, *unguiculata*, and *exilis* occupy pine-oak woodland habitats, grasslands, and disturbed roadside habitats. Although there is a narrow contact zone in the eastern portion of *xantiana*’s range and the western part of *parviflora*’s (where the subspecies occupy similar habitats), *parviflora* also occurs in arid juniper-pinyon woodlands and dry shrublands in the eastern portion of its range.

### 3.2. Experimental Design of Field Study

In spring 2008, we evaluated pollen performance in a total of eight field populations (2 per taxon) near Lake Isabella (Kern and Tulare Counties, CA, USA). Additional information about each population, including sampling dates and GPS coordinates, can be found in Supplementary Matirials Table S1. In each population, we haphazardly sampled 18–22 individuals to serve as pollen donors; using their pollen, we evaluated pollen performance following hand-pollination of recipient flowers on other individuals in the same population. Pollen from each pollen donor was applied to a maximum of 15 receptive stigmas [(1–4 recipient flowers per plant × 3 maternal plants were used for outcrossing) + 1–3 self-pollinations]. 

To ensure that each recipient flower was free of pollen before administering hand pollinations, *unguiculata* and *xantiana* flowers were emasculated (the anther sacs were removed) after they opened, but before stigmas became receptive. Often, stigmas of *exilis* and *parviflora* were receptive and in direct contact with dehiscent anthers as the flowers opened. To prevent self-pollination in these taxa, *flowers were emasculated while in the bud stage. Following emasculation*, *in all four focal taxa*, *1–2 days* prior to the hand-pollination of the recipients’ flowers, we covered the pre-receptive, emasculated recipient flowers or buds with a an approximately 5 cm segment of a hollow, partially transparent, perforated plastic cylinders (10 mm in diameter, “boba” straws) to prevent self-pollination and pollen deposition by visiting insects. The pores in each cylinder were large enough to allow air to enter, but excluded insect visitors; the top and bottom openings of the cylinders were loosely packed with cotton to exclude insects. The cylinders were removed immediately prior to hand-pollinations, which were performed on receptive, virgin stigmas by applying a single, clump-free, layer of fresh pollen to receptive floral stigmas with a blunted dissecting probe. To maximize consistency in the number of pollen grains deposited on stigmas, one person, wearing jeweler’s magnifying glasses, performed all pollinations for this study. Following hand-pollination, cylinders were placed on each flower to prevent additional pollen deposition by insects. Recipient flowers on each maternal plant were labeled with a permanent marker on the node adjacent to the flower. Each mark corresponded to positional data recorded at the time of pollination (the position of the flower on the primary stem or on a lateral branch) in order to indicate pollen donor identity and the exact time of pollination. The time elapsed between pollen collection and supplemental pollination was always less than 20 min. After allowing pollen tubes to grow for exactly 2.5 hours, whole flowers were excised from the plant and fixed in formalin-acetic acid (FAA) to arrest pollen tube growth. Flowers were then brought to the laboratory for style excision and pollen tube visualization. To remove individual styles, we first used a dissecting microscope to identify the base of each style. Forceps were then used to gently detach styles from the top of the ovary. Excised styles were stored in FAA-filled 1.5 mL microcentrifuge tubes prior to additional processing. 

Each maternal plant generally received pollen from multiple pollen donors and we aimed to evaluate each donor’s pollen performance on multiple flowers per maternal plant. The actual number of recipient flowers per plant, however, varied due to inter-population differences in both population size and the number of flowers with receptive stigmas. For example, *exilis* populations were small in size and were composed of fewer individuals than *unguiculata*, *parviflora*, and *xantiana* populations. Moreover, individual *exilis* plants tended to be small; many bore fewer than two receptive stigmas at a time. In *exilis* populations, we attempted to locate maternal plants bearing multiple female-phase flowers; this, however, was not always possible. When maternal plants bearing multiple flowers could not be located, one flower per plant was pollinated; this was necessary for 13 out of the 73 *exilis* maternal plants used in this study. Self-pollinations were not performed at the Willow Spring *exilis* population due to logistical constraints.

Pollen tube kinetics are well-known to be influenced by temperature [57,59,60,62]. Because the goal of this study was to compare pollen performance traits between sister taxa with contrasting mating systems, we aimed to account for the effects of differences in ambient temperature within populations and among populations at the time of sampling on pollen performance. We recorded air temperature next to each flower at the time of pollination and at the time of style harvest (°C) with a digital thermometer. See the *Statistical Analysis* section below for a detailed description of how we controlled statistically for the effect of temperature on pollen performance in the style prior to comparing sister taxa.

### 3.3. Pollen Tube Growth

In the laboratory, we used methods similar to those described by Martin [88] to visualize pollen tubes of all four focal taxa. Excised styles were soaked for 28–40 hours in 8 M NaOH, rinsed in distilled water, and then stained for 2–4 hours in a 0.1 N K_2_HPO_4_ solution with 0.1% aniline blue dye. After staining, calipers were used to measure each style’s length to the nearest 0.5 mm. Styles were then squashed and mounted onto individual microscope slides. This process allowed the visualization, counting, and measurement of pollen tubes using an epifluorescence microscope (Olympus BX61) equipped with a DAPI excitation filter. 

As individual *Clarkia* pollen tubes travel through the style, callose plugs, which ensure that cytoplasmic contents remain contained within the leading tip of the tube, are deposited in a regular manner roughly every 1.0 mm. Individual pollen tubes can be difficult to visualize using Martin’s staining method; young pollen tubes generally do not produce enough sugar to stain clearly. We therefore measured pollen tube lengths by counting the number of callose plugs, which stain brightly in the presence of aniline blue, within each 1.0 mm interval along the entire length of each style (see [34] for additional detail regarding the use of this method for estimating PTGR). We then assumed that pollen tubes ending within a given 1.0 mm interval stopped halfway through that interval. For example, we assumed that any tubes that did not grow beyond the first 1.0 mm of the style stopped growing after 0.5 mm. This allowed us to calculate an estimate of mean pollen tube length achieved 2.5 hours after each pollination event (Table 1). We then estimated pollen tube growth rates (PTGR, mm * hour^−1^) based on the amount of time that elapsed between pollination and style excision. We also estimated the average proportion of the entire style length traversed by pollen tubes per hour. To meet assumptions of normality and homoscedasticity, data were log_10_ transformed (PTGR) or arcsin-square root transformed (proportion of the style length traveled) prior to statistical analysis. 

### 3.4. Pollen Performance Traits

We assessed three measures of pollen performance on each style harvested. Pollen germination was scored as a categorical variable indicating whether or not pollen tubes had penetrated the style. Because ungerminated pollen grains often washed off of stigmatic surfaces during the fixation and staining process, the percentage of all initially-deposited pollen grains that germinated could not be reliably estimated. Instead, we concluded that pollen germination had occurred if at least one pollen tube was observed at the stigma-style junction 2.5 hours post-pollination. We also estimated PTGR (the mean speed by which pollen tubes travel through the style, see above), as well as the mean proportion of the entire style length traveled per hour.

### 3.5. Statistical Analysis

#### 3.5.1. The Association between Taxon Identity and Pollen Germination

On multiple occasions, we observed that no pollen tubes had entered the styles, generally because pollen grains failed to germinate. This observation was especially common following both cross- and self-pollination of *exilis* flowers. We used two-tailed Fisher’s Exact Tests to determine whether the frequency of pollen germination differed statistically between the selfing and outcrossing sister taxa (*exilis* and *unguiculata*, as well as *parviflora* and *xantiana*). Because the pollen source (self or cross) may influence pollen germination and growth, pollen germination following cross- and self-pollinations was evaluated separately. 

#### 3.5.2. Accounting for the Effect of Temperature on Pollen Tube Growth through Styles

**Figure S1:**
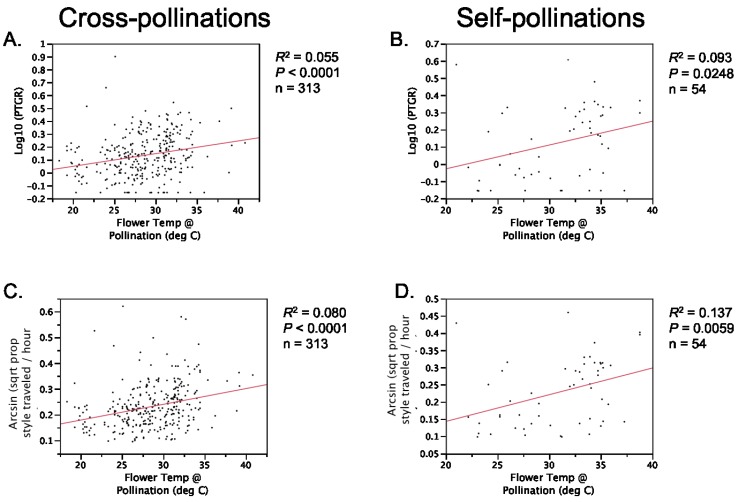
Relationships between temperature and pollen performance traits in *exilis* and *unguiculata.* (**A**) pollen tube growth rate (PTGR) following cross-pollination, (**B**) PTGR following self-pollination, (**C**) proportion of the style traveled following cross-pollination, (**D**) proportion of the style traveled following self-pollination.

**Figure S2:**
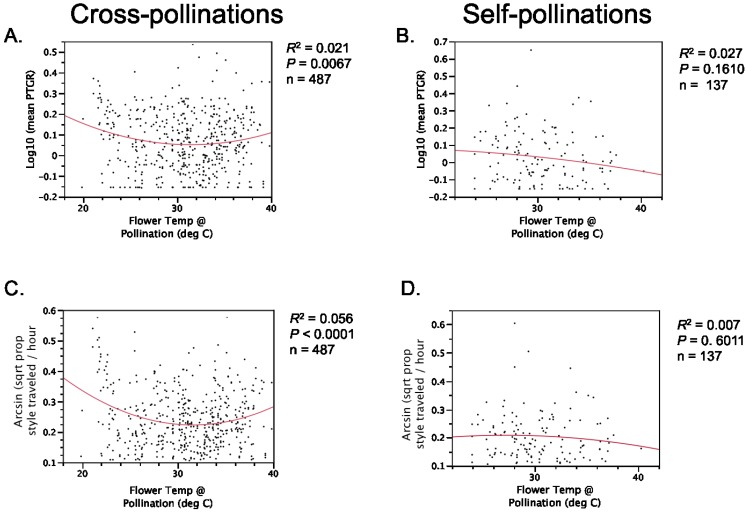
Relationships between temperature and pollen performance traits in the selfer *parviflora* and the outcrosser *xantiana*. (**A**) pollen tube growth rate (PTGR) following cross-pollination, (**B**) PTGR following self-pollination, (**C**) proportion of the style traveled following cross-pollination, (**D**) proportion of the style traveled following self-pollination.

To control statistically for the effect of temperature on pollen tube growth through the style in each pair of sister taxa, we regressed PTGR and the proportion of the style length travelled on temperature at the time of pollination (°C). For each pair of sister taxa, these regressions were performed after pooling data from the two taxa. C*ross-taxon residuals*, which were subsequently used to compare each pollen performance trait between sister taxa, were then calculated. For *unguiculata* and *exilis*, we conducted linear regressions in which a given measure of pollen performance (PTGR or the proportion of the style length traveled) was regressed on temperature at the time of pollination (°C). See Supplementary Materials Figure S1 for bivariate plots and *r^2^* values associated with these models. For *xantiana* and *parviflora* we used residuals estimated from polynomial regressions in which a given measure of pollen performance (PTGR or the proportion of the style travelled) was regressed on temperature at the time of pollination and (temperature at the time of pollination)^2^; polynomial regressions provided a better fit than the linear regressions for these taxa. Figure S2 presents the bivariate plots and *r^2^* values associated with these models. For each pair of sister taxa and for measures of pollen tube growth through the style, we calculated two sets of temperature-adjusted *cross-taxon* residuals: one for cross-pollinations and one for self-pollinations. The residuals calculated from these models were used in the ANOVAs described below. Because we aimed to compare sister taxa with respect to the pollen tube growth of viable pollen grains through the style, zero values, which were associated with pollen grains that did not germinate or whose tubes failed to enter the style 2.5 hours post-pollination, were excluded from this analysis. See Table S2 for (non-temperature-adjusted) mean pollen performance traits and associated standard errors.

#### 3.5.3. Pollen Tube Growth through the Style: Cross-Taxon Comparisons

For each type of pollination (cross or self), we used mixed-model analyses of variance (ANOVAs) to compare sister taxa with respect to their mean temperature-adjusted PTGR and the proportion of the style length traversed. These models included the following three effects: Taxon (fixed effect), Pollen Recipient nested within Taxon (random effect), and Pollen Donor nested within Pollen Recipient and Taxon (random effect). We conducted these analyses (and report them in Table 3, Table 4) after examining the results of four-factor mixed model nested ANOVAs, which included Taxon (fixed effect), Population nested within Taxon (random effect), Pollen Recipient nested within Population and Taxon (random effect), and Pollen Donor nested within Pollen Recipient, Population, and Taxon (random effect). These mixed models indicated that pollen performance did not differ statistically between populations of the same species or subspecies following cross- or self-pollinations, so we excluded the Population effect from the model reported here. For all mixed model ANOVAs, we used the Sattherthwaite method to estimate degrees of freedom (restricted maximum likelihood, REML).

To determine whether sister taxa differ with respect to *phenotypic variation among pollen donors*, we used the temperature adjusted *cross-taxon* residuals to test the hypothesis that pollen performance trait variances differ among populations of sister taxa. Within each population, we first calculated each pollen donor’s mean PTGR and mean proportion of the style length traveled across all recipient flowers following cross-pollinations. To estimate each population’s trait means, we then calculated the mean of the donors’ means for each pollen performance trait. To evaluate population-level phenotypic variation among donors, trait variances were calculated as the variance among the donor means. We compared these variances among populations of sister taxa to determine whether any single population exhibited higher or lower variation in pollen performance traits than the other populations. The default tests in JMP 9.0 (O’Brien, Brown-Forsythe, Levene, and Bartlett tests) were used to test for heterogeneity of trait variances among populations of sister taxa. If differences were detected by these tests, we used the analysis of means for variance test (Levene’s ANOM for variances) to identify populations whose absolute deviations from the median differed from the average cross-group absolute deviation from the median.

#### 3.5.4. Pollen Performance following Cross- and Self-Pollination in Each Focal Taxon

Within each taxon, we compared three measures of pollen performance (pollen germination, PTGR, and the proportion of the style length traveled) following self- *versus* cross-pollinations. Fisher’s Exact Tests were used to evaluate the association between pollination type (cross or self) and the relative frequencies of styles on which pollen germination did or did not occur in *unguiculata*, *exilis* (Stark Creek population only), *xantiana*, and *parviflora*.

**Figure S3:**
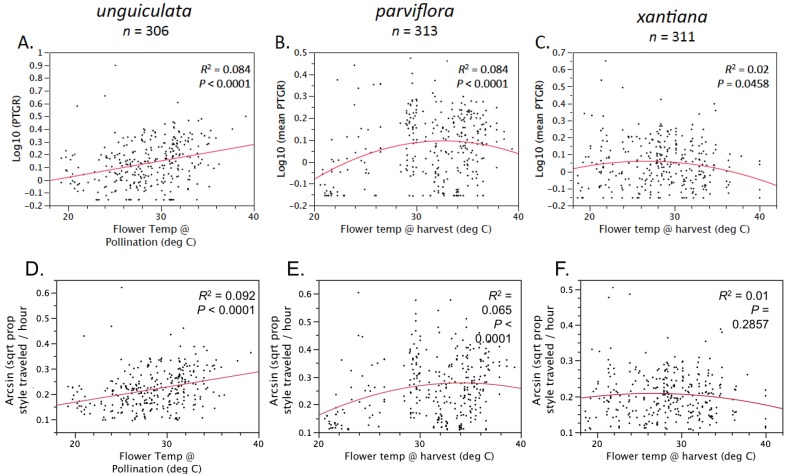
Relationships between temperature and pollen tube growth rate (PTGR) (**A-C**) and the proportion of the style traveled per hour (**D-F**) in *unguiculata, parviflora* and *xantiana*. These models were used to calculate the *taxon-specific* residuals that were used to compare pollen performance following cross- and self-pollinations.

**Figure S4:**
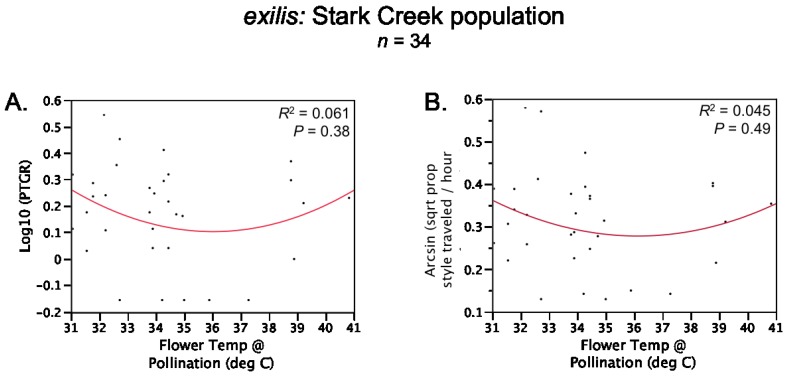
Relationships between temperature and (**A**) pollen tube growth rate (PTGR) and (**B**) the proportion of the style traveled per hour in the Stark Creek population of *exilis*. These models were used to calculate the *taxon-specific* residuals that were used to compare pollen performance following cross- and self-pollinations.

We calculated *taxon-specific* residuals to compare traits associated with pollen tube growth following self-and cross-pollinations, taking into account the effect of temperature within each focal taxon [*unguiculata*, *exilis* (Stark Creek population only), *xantiana*, and *parviflora*]. The objective of these analyses was to assess the effect of pollination type on PTGR and the proportion of the style length travelled by viable pollen grains. Therefore, zero values, which were associated with pollen grains that did not germinate or whose tubes failed to enter the style 2.5 hours post-pollination, were excluded from these analyses. Temperature-adjusted performance traits for *unguiculata* were calculated using the residuals from linear regressions in which a given pollen performance trait (PTGR or the proportion of the style traveled) was regressed on temperature at the time of pollination (°C). For *xantiana* and *parviflora* we used the residuals from polynomial regressions in which a given measure of pollen performance (PTGR or the proportion of the style travelled) was regressed on (temperature at style harvest) and (temperature at style harvest)^2^ because these regressions gave a better fit than the linear regressions. See Figure S3 for bivariate plots and *r^2^* values associated with these models. For the Stark Creek *exilis* population, we controlled statistically for the effect of temperature by using the residuals from polynomial regressions in which PTGR or the proportion of the style traveled was regressed on temperature at the time of pollination (°C) and (temperature at the time of pollination)^2^. See Figure S4 for bivariate plots and *r^2^* values associated with these models. For each taxon, variance components associated with the random effects of Pollen Donor and Pollination Type nested within Pollen Donor were estimated using the REML method for each temperature-adjusted pollen performance trait. For *unguiculata*, *xantiana*,and *parviflora*, we conducted these two-factor analyses after initial models including three random effects (Pollen Donor, Pollination Type nested within Pollen Donor, and Population) indicated that pollen performance did not differ significantly between populations of the same species or subspecies. The 95% confidence intervals spanning the variance components associated with the Population effect included zero. All analyses were performed using JMP 9.0 (SAS Institute).

## 4. Conclusions

Differences between selfers and outcrossers in the diversity of potential pollen donors that arrive on stigmas have been predicted to drive the evolutionary divergence of sexually selected traits, such as the speed by which pollen grains germinate and travel through the style. Here we demonstrate that pollen performance differs under field conditions between *Clarkia* *exilis* and its outcrossing sister species, *C*. *unguiculata*, in a manner that is consistent with the hypothesis that pollen competition promotes the evolution of rapid pollen performance in outcrossing taxa relative to their selfing counterparts. Plants from *unguiculata* populations exhibited faster or more frequent pollen germination than plants from *exilis* populations; *unguiculata* pollen tubes were more likely to have entered the style within 2.5 hours of pollination. Surprisingly, we found that *C. xantiana* ssp. *parviflora* exhibited faster PTGR and greater proportional distance travelled within the style than its outcrossing counterpart, *C. xantiana* ssp. *xantiana*. These contrasting patterns suggest that gamethophytic evolution following mating system transitions is likely to involve a variety of genetic and ecological factors. The extent to which the evolution of pollen performance traits is influenced either by local environmental conditions or by strong correlations with plant traits other than mating system in the *Clarkia* taxa studied here, however, merits further investigation. Case studies evaluating additional pairs of sister taxa are needed before generalizations regarding the joint evolution of mating system and pollen performance can be made. Nevertheless, the contrasting patterns that we observed in the two pairs of *Clarkia* sister taxa are likely driven in part by differences in the magnitude of gametophytic competition in *unguiculata* and *xantiana* populations. In populations characterized by consistent pollen limitation, such as *xantiana*, selection on pollen performance traits may be weak or neutral. Consequently, this study highlights the importance of evaluating the joint evolution of pollen performance and mating system in field populations, where abiotic and biotic ecological factors may influence the phenotypic expression of and ultimately the evolution of sexually selected traits. 

## Supplementary Materials

**Table S1:** Location, elevation, and sampling information for populations in which pollen performance was evaluated in 2008.

Taxon	Population Name	Elevation (m)	GPS coordinates	Sampling Dates, 2008	# Flowers Pollinated
*exilis*	Stark Creek	443	35°28.27' N 118°43.55' W	April 26	138
	Willow Spring	365	35°40.23' N 118°54.13' W	April 22, April 25	62
*unguiculata*	Live Oak	430	35°28.81' N 118°44.89' W	May 9, May 12	197
	Jack and Stage	1006	35°47.75' N 118°42.16' W	June 3	157
*parviflora*	Long Valley	1607	35°48.54' N 118°05.34' W	June 10	176
	Wofford Heights	859	35°41.36' N 118°28.00' W	May 11	160
*xantiana*	Borel Road	707	35°35.04' N 118°31.30' W	June 2	157
	Camp 3	896	35°48.69' N 118°27.70' W	May 21	194

**Table S2:** Non-temperature adjusted means and standard errors associated with pollen performance traits in all four focal taxa following cross- and self-pollinations. Styles in which no pollen tubes were observed at the stigma-style junction were not used to generate these values.

			Pollen Tube Growth Rate (PTGR, mm hour^−1^)			Proportion of the style length (traveled per hour)	
Pollination type	Taxon	N	Mean	SE		Mean	SE
Cross	*exilis*	55	0.9988	0.0928		0.0923	0.0092
	*unguiculata*	259	0.9613	0.0424		0.0516	0.0022
	*parviflora*	273	0.7657	0.0266		0.0789	0.0035
	*xantiana*	240	0.6150	0.0277		0.0409	0.0019
Self	*exilis*	7	0.9876	0.2333		0.0877	0.0208
	*unguiculata*	47	0.9945	0.1148		0.0550	0.0063
	*parviflora*	54	0.4856	0.0653		0.0442	0.0078
	*xantiana*	94	0.6452	0.0519		0.0421	0.0033
